# A short history of heme dioxygenases: rise, fall and rise again

**DOI:** 10.1007/s00775-016-1412-5

**Published:** 2016-12-01

**Authors:** Emma L. Raven

**Affiliations:** Department of Chemistry, University of Leicester, University Road, Leicester, LE1 7RH UK

## Abstract

It is well established that there are two different classes of enzymes—tryptophan 2,3-dioxygenase (TDO) and indoleamine 2,3-dioxygenase (IDO)—that catalyse the O_2_-dependent oxidation of l-tryptophan to *N*-formylkynurenine. But it was not always so. This perspective presents a short history of the early TDO and IDO literature, the people that were involved in creating it, and the legacy that this left for the future.

## Power to the people

There are fashions in science, just as there are in styles of trousers. Fashions in science are influenced by variables large and small: governments that can control the political climate; policy and funding streams; universities and other institutions that control scientific appointments; geography that can enhance or restrict access to ideas or technology; and the rate of development of technology itself which can either slow down or suddenly speed up scientific progress. But more often than not, fashions in science are also influenced to a greater or lesser extent by people, for it is the people who create the focus, the scientific stimulus, and the new ideas upon which future progress must be based.

In the case of the heme dioxygenase enzymes, a handful of people were highly influential and they laid the foundations for the development of the area over the next 60 years. This short perspective summarises these and other early contributions to the heme dioxygenase field.

## In the beginning there were two

As often happens, two people drew more or less the same conclusions at more or less the same time. In 1955, Mason [1] and Hayaishi [2, 3] independently proposed that enzymatic incorporation of molecular oxygen into a substrate was possible. At the time, this was an almost unthinkable idea—probably because the prominent German chemist and Nobel Prize winner Heinrich Wieland (and naturally, therefore, almost everybody else) had ruled the possibility out—but this did not stop Mason and Hayaishi thinking about it quite a lot.

**Fig. 1:**
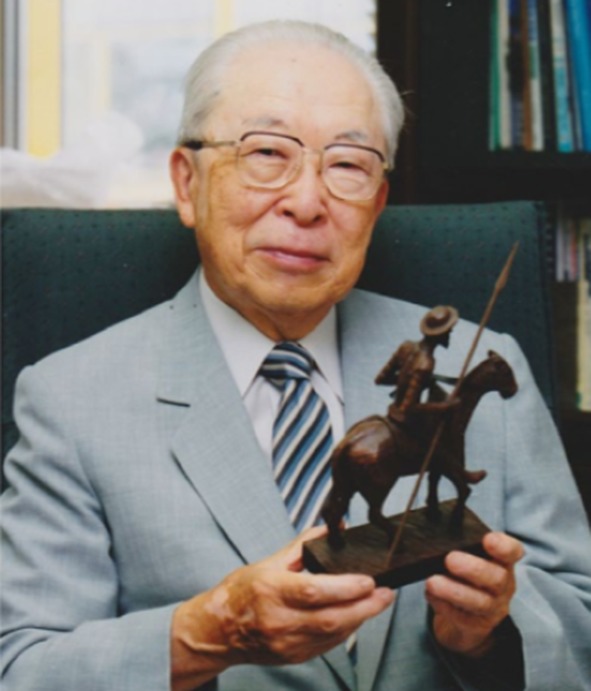
Professor Hayaishi pictured holding a model of the fictional hero Don Quixote, of whom he was a long-standing admirer (see [113]). The photograph was provided by Hayaishi’s daughter, via his former secretary, to Prof. Masao Ikeda Saito

Mason’s experiment was published in 1955 [4] and led to his now famous classification of enzymatic oxygen metabolism [5]. Mason proposed that two atoms of molecular oxygen can be incorporated into the substrate and he termed this type of activity an “oxygen transferase”. Hayaishi, using mass spectrometry, demonstrated quantitative incorporation of ^18^O_2_ (and, importantly, not H_2_^18^O) into the substrate in the pyrocatechase reaction [6]. He too referred to the activity as “oxygen transferase”. Hayaishi, Fig. 1, later introduced the term “oxygenase” to the literature [7], a proposal that had first been mooted at an ACS meeting in 1956 [8] and which has stuck in the heme literature ever since.

**Fig. 2:**
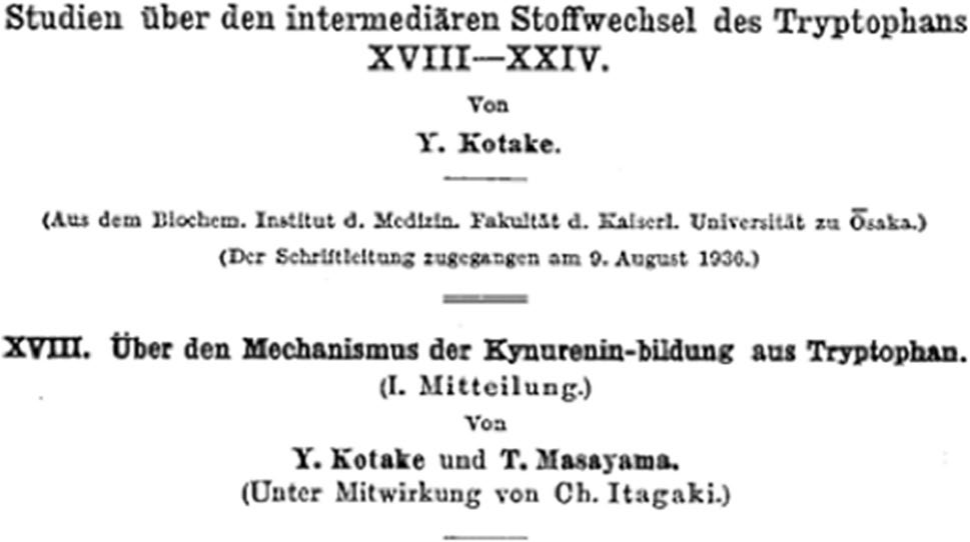
One of the seminal (but for some readers somewhat impenetrable) papers from Kotake [10]

## Where there’s muck there’s brass

**Fig. 3:**
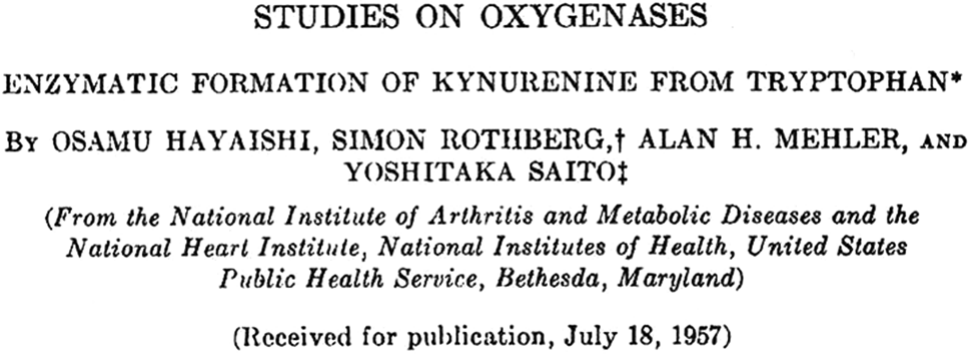
Hayaishi’s seminal paper [11] reporting that both atoms of oxygen incorporated into the product during tryptophan oxidation are derived from ^18^O_2_. Reproduced with permission from The American Society for Biochemistry and Molecular Biology

**Scheme 1:**
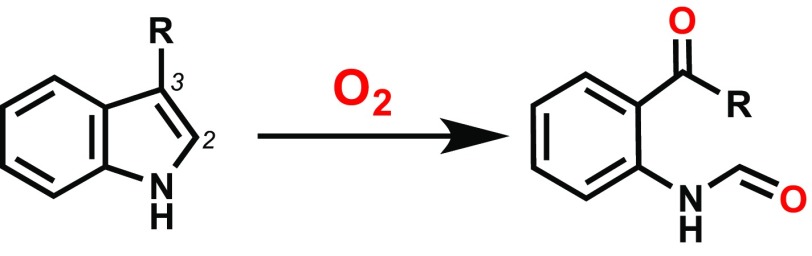
The oxidation of tryptophan to NFK, as catalysed by IDO and TDO

Hayaishi’s introduction to tryptophan metabolism had occurred from a chance encounter at Osaka University with Kotake. Kotake had devoted much of his life’s work to the biochemistry of that particular amino acid in animals and had published some of the earliest seminal studies in the 1930s [9, 10], Fig. 2. Japan at that time was in the aftermath of the war, and Osaka had been totally demolished. Kotake, perhaps wishing to see the tradition of a Japanese effort in the tryptophan area continued into the future, donated several grams of the precious compound to Hayaishi. With no chemicals, no equipment to speak of, a non-existent consumables bud get, no animals and probably no students either, Hayaishi has pointed out [2] that his options were somewhat limited. By necessity, he went outside and, literally, dug up some muck and mixed it with his compound. From there he was able to demonstrate that certain microorganisms in soil can grow using tryptophan, and what followed was a series of four consecutive papers all looking at enzymatic incorporation of O_2_ into a substrate [7, 11–13]. One of these, Fig. 3 [11], concerned itself with the oxidation of tryptophan and examined the conversion of tryptophan to *N*-formylkynurenine (NFK) in *Pseudomonas* extracts using mass spectrometry, Scheme 1. It was the first demonstration that “…both atoms of oxygen incorporated in the oxidative step are derived from oxygen gas but not from water” [11].

At that time, the metabolism of tryptophan was just beginning to be clarified, and several people—including the distinguished A. Neuberger from Mill Hill in London^1^ [14, 15]—had come to the conclusion that NFK was part of the process. But the enzyme responsible for the activity had not been fully established, and it had been temporarily denominated as a “tryptophan peroxidase”. The early nomenclature, to put it mildly, would send shivers down the spine of an IUPAC committee. A list of terms as long as the Royal Mile appeared in print: tryptophan pyrrolase (which still pervades in the literature), tryptophan peroxidase, tryptophan oxidase, tryptophan peroxidase-oxidase, and tryptophan oxygenase were all used (see for example [14, 16–22]). Most authors evidently found the process of deciding between these terms to be an impossible task and so used them all at the same time. It was Hayaishi himself who brought some order to the confusion, by suggesting in 1970 [23] that the enzyme would most sensibly be named tryptophan 2,3-dioxygenase (TDO), to distinguish its reactivity from any other enzymatic tryptophan activity (e.g. in the formation of tryptophan 5-monooxygenase). Even so, it took some years before the literature adjusted to this brave new world in which one enzyme had only one name.

It had been known at this time that there were other enzymes from different sources capable of catalysing the same reaction as TDO, but with much less substrate specificity than TDO. As far back as 1967, Hayaishi had identified one such enzyme from rabbit intestine [17] and it was initially identified as “tryptophan pyrrolase (tryptophan 2,3-dioxygenase)”. In view of the broad substrate specificity of these other enzymes, it was suggested [24], again by Hayaishi, that they be designated as indoleamine 2,3-dioxygenases (IDO), to differentiate them from the TDOs (which are specific for tryptophan) and to convey the message that other substituted indoles were also accessible by these enzymes. Although even as late as 1974 the community was still afflicted by chronic indecision on the names for their pet enzymes, as the early proposal [24] also suggested the very awkward and certainly confusing “indoleamine 2,3-dioxygenase (pyrrolase)” nomenclature. But by the end of the 1970s the literature was more consistent, with regular papers describing the properties of the now easily recognisable indoleamine 2,3-dioxygenase enzyme (see for example [25–34]).

In the intervening years, a much clearer picture has emerged. It is now well known that the IDOs and the TDOs, whilst catalysing the same reaction, have slightly different properties. IDOs are monomeric, while the TDOs are tetrameric. IDOs have wide substrate specificity and will oxidise a range of indoleamine derivatives, while the TDOs are much more discriminating and typically oxidise only l-Trp at any respectable catalytic rate. Also, while IDO is widely distributed in all tissues but not the liver, TDO has most often been cited as being found only in the liver (although there is emerging evidence that it is also located in some cancer cells [35]).

## The 1970s: the emergence of heavy metal

**Fig. 4:**
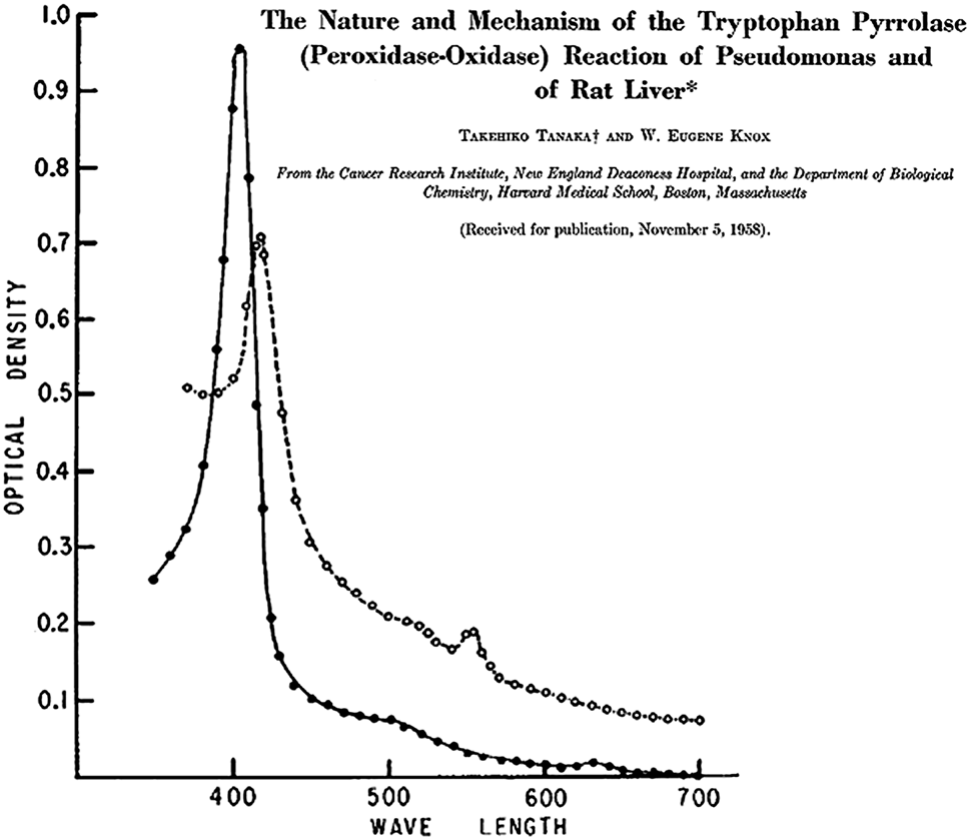
An early UV–visible spectrum of TDO [16], showing a Soret absorbance at around 405 nm (note the nomenclature for the name of the enzyme). Reproduced with permission from The American Society for Biochemistry and Molecular Biology

The idea that there could be a role for a metal in tryptophan oxidation took a while to sink in. The earliest mention of a heme dependency that this author was able to identify came in 1959 (and there were indications even earlier than that [36]). Tanaka and Knox [16] presented UV–visible spectra for the TDO from rabbit liver, Fig. 4, with Soret bands that are surprisingly close to those found for recombinant mammalian TDOs and bacterial TDOs isolated many decades later [37–43], and they suggested a similarity with the by then well-known ferrous oxy hemoglobin system. A series of papers from Feigelson going back as far as 1961 also demonstrated very fluently that the activity of TDO was dependent on heme (see for example [20, 21, 44–47]). By the late 1970s, the role of heme had finally become “mainstream” in the IDO literature as well [29–34].

The suggestion [22, 48] that copper was involved in TDO catalysis turned out not to be correct [49, 50], but nonetheless generated heated debate.

## The 1980s onwards

**Fig. 5:**
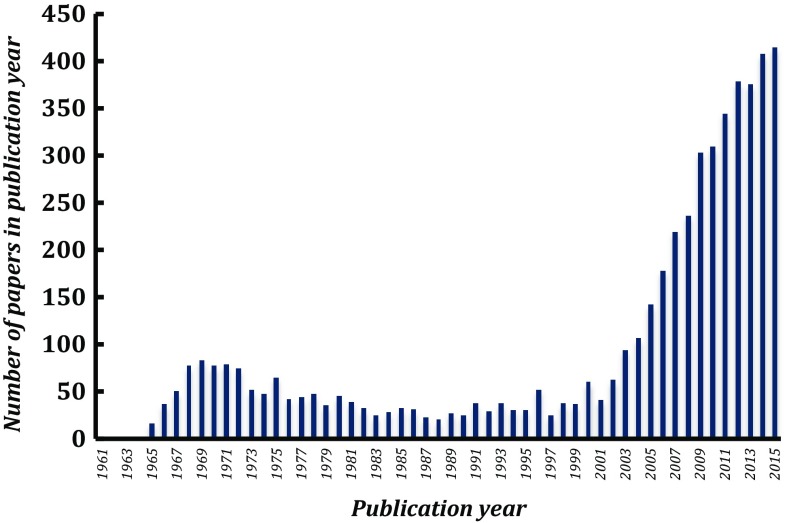
An analysis from Web of Science showing the total number of literature citations in each year when searching by title in Scopus for indoleamine 2,3-dioxygenase or tryptophan 2,3-dioxygenase, going back to 1960

In the 10 years or from 1980, after the extensive work that had been done previously (as summarised above), a large volume of spectroscopy and kinetic work appeared on both IDO and TDO. This has been comprehensively summarised in an outstanding review by Sono and Dawson in 1996 [18] and will not be rehearsed here again. But an analysis of the literature, Fig. 5, shows that there was a lull in publication activity around the late 1980s and early 1990s. The field stalled to some extent, waiting for the development of suitable systems for expression of IDO and TDO in *E. coli.* An early report [37] of expression of rat TDO in *E. coli* stood out and led the way as it preceded, by some margin, the publication of numerous other expression systems for TDO/IDO in mammalian [38–40, 51–60], bacterial [61–63], insect [64–66], fungal [67, 68], yeast [67] and other [69] systems.

## A new dawn from 2000: arise again

The Dawson review was very timely, because it included a focused but detailed summary of all of the previous IDO and TDO work. With expression systems emerging soon afterwards (see above), the review set the scene for a resurgence in interest in these enzymes over the next two decades, Fig. 5. Mauk has referred to this as a “renaissance” [70]. Much of the new work in the last few years has been motivated by the search for IDO inhibitors relevant to therapeutic application in cancer [71–73].

### Structure

In terms of functional analyses, there have been some substantial developments since 2000 (see also previous reviews [74–76]). Of special note is the landmark human IDO structure from Sugimoto and Shiro [52], which gave the first glimpse of the highly hydrophobic IDO active site in complex with the inhibitor 4-phenylimidazole bound to the heme; other structures in complex with related inhibitors have recently appeared [77, 78] and form an important structural framework for structure-based drug design in the future.

The structure of the *X. campestris* TDO in complex with tryptophan [61], and other TDO structures have also been important [62, 66]. The structure of human TDO in the apo form (i.e. without heme bound) has also been reported [79]. There are no structures for inhibitor-bound TDOs, with structure-based virtual screening providing the best information so far [80]. It has been suggested from spectroscopic work that the heme sites in (tetrameric) TDO may not be equivalent [81]. The recent structure of human TDO in complex with both O_2_ and l-Trp [82] is another step forward, and allows the first reliable visualisation of the binding orientation in the ternary complex.

There is evidence, at least in IDO, that the active site and other regions of protein structure that are not visible in the X-ray maps are conformationally mobile and that this might affect reactivity [83]; similar flexibility is known to be important in the P450_cam_ system (see for example [84–86]).

### Mechanism

Techniques other than crystallography have been needed to make progress on mechanism, and there is much work to do yet before the mechanism is fully clarified. Early proposals for the mechanism of NFK formation [87] have been substantially revised in recent years. The generational echoes have resonated loudly, as some of the newer ideas on mechanism [88] were derived from mass spectrometry experiments (as in the early days [6]).

**Fig. 6:**
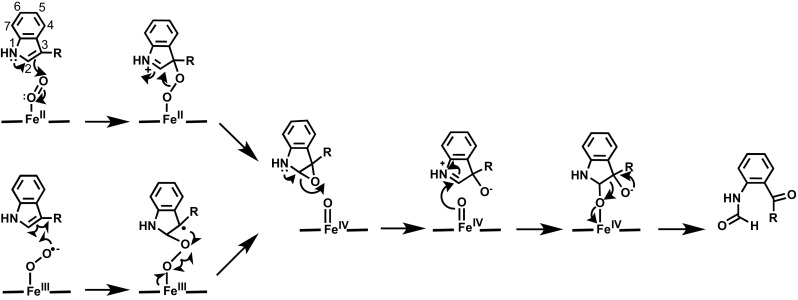
A mechanism for tryptophan oxidation, consistent with all of the recent observations. Electrophilic addition (*top*) and radical addition (*bottom*) are possible. See text for details. Recent structural information [82] indicates that NFK is bound to the iron in the enzyme–product complex

Spectroscopy and kinetics, at one time the poor relations compared to the mighty crystallography, are now playing a leading role again just as they did in the 1980s (including recently on indoleamine 2,3-dioxygenase 2 (IDO-2) [89]). In terms of mechanism, there seems to be a consensus emerging that the mechanism outlined in Fig. 6 is reasonable, but things are far from being conclusively established and, bearing in mind the early mechanistic red herrings in this area [87], caution is still needed. Computational approaches have proved very useful in elucidating the mechanism [90–93].

Early proposals [87] for tryptophan oxidation suggested a base-catalysed abstraction mechanism and no change in oxidation state of the metal, but several groups had independently reported [42, 88, 94] that the 1-Me-l-Trp analogue was also reactive, and it was noted [95] that this is not consistent with a base-catalysed abstraction mechanism. Mutational data where the presumed active site base (histidine) had been removed were also not consistent with base-catalysed abstraction [96]. Two other mechanisms, Fig. 6, have been put forward [88, 90, 91, 97], but there is little in the way of firm evidence for either. Electrophilic addition from the ferrous oxy species, Fig. 6, is one possibility: recent evidence in TDO [98] (using modified hemes that were first used more than 30 years ago [99]) supports this. We have noted [74, 75] that oxygen may not be an especially good electrophile if it is bound to the heme as a ferric superoxide species, and there is spectroscopic evidence for a ferric superoxide species [97] from Raman’s work. An alternative suggestion [97] is radical addition from the ferric superoxide, Fig. 6 (bottom). Both pathways lead to formation of a ferryl (Fe^IV^) species. There is mass spectrometry evidence for epoxide formation [100], but later intermediates in the mechanism are not clarified. Addition of oxygen across either the C^2^ or the C^3^ position of the substrate is possible for both the radical and electrophilic mechanisms, and at present this is a moot point. Both possibilities have been suggested [82, 88, 90, 91, 93, 97].

**Fig. 7:**
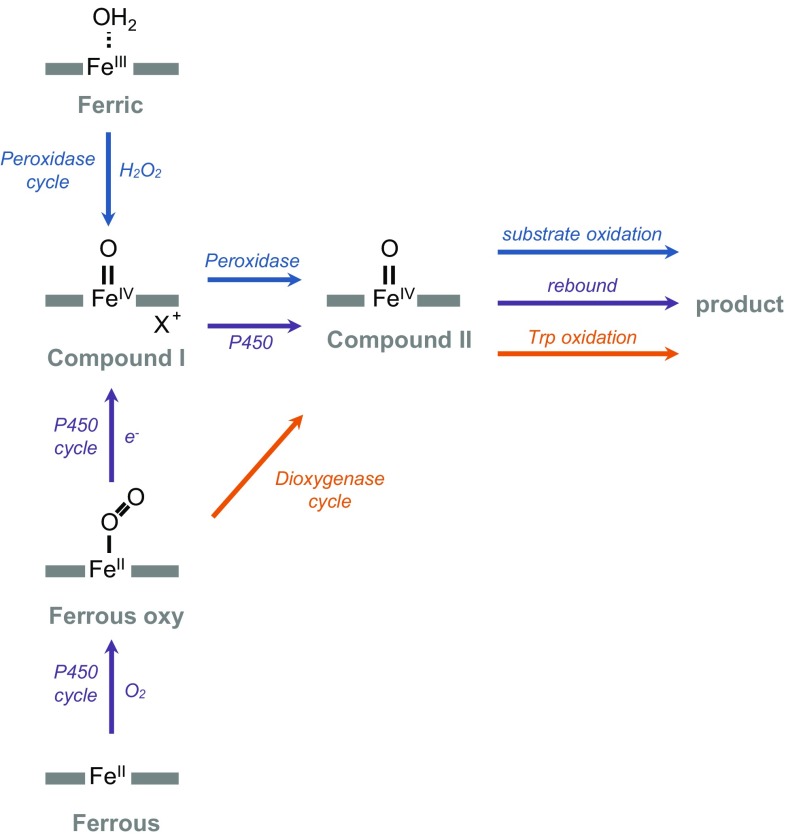
A comparison of mechanisms of oxygen activation in different heme enzymes. The well-known peroxidase mechanism (*blue arrows*) goes via ferric heme directly to Compound I and then to Compound II by one electron oxidation of substrate [114]. The P450s (*purple arrows*) use the same Compound I species but they access it through the ferrous oxy species by one electron reduction, and by rebound mechanisms access the same Compound II species [115, 116]. The identification [97, 101, 102] of a Compound II species in IDO (which accumulates in the steady state) aligns the dioxygenase mechanism (*orange arrows*) with these established patterns of reactivity in other heme systems. It has been assumed that IDO and TDO react by the same mechanism, but Compound II in TDO has never been detected in the steady state. There is evidence that the absence of Compound II in the steady state in TDO might be due to a change in the rate-limiting step in TDO compared to IDO, such that Compound II does not accumulate [117]. Note that there is also evidence [118] that IDO can exhibit indole peroxygenase activity (i.e. a peroxide-dependent insertion of oxygen into indole), similar to the well-known peroxide shunt of the P450s

A real step forward was made using resonance Raman [97, 101] to identify a ferryl (Compound II) intermediate in the IDO mechanism. The same Compound II species has recently been identified kinetically and is also observed during oxidation of 1-methyl-l-Trp and a number of other substrate analogues [102], providing strong evidence that IDO uses the same mechanism for oxidation of tryptophan as it does for oxidation of other substrate analogues. We have argued [74, 75, 103] that since the process of oxygen activation in most heme enzymes (e.g. P450s, peroxidases, etc.) is also achieved through formation of highly oxidised iron intermediates, this brings the dioxygenases into line with the oxidative mechanisms used in other heme enzymes, as illustrated schematically in Fig. 7. One difference in the dioxygenases is that continuous re-reduction of an oxidised ferryl heme (through an associated reductase) is not required, because all of the available evidence indicates that the dioxygenases only require a single, initiating reduction of ferric heme. The reader is referred to previous reviews [74, 75, 103] for a fuller discussion.

### Substrate binding and catalysis

It had been noted from very early on [17, 104] that the rate of tryptophan turnover in IDO decreases at high concentrations of substrate. This was originally proposed [104] to be a consequence of substrate binding to the ferric form of the enzyme, but this is not consistent with the known [51, 105] increase in reduction potential on substrate binding and has therefore been questioned [106]. Some evidence suggests that the sequence of binding of O_2_ and the substrate at high and low substrate concentrations is important [106–108], possibly linked to changes in the reduction potential on substrate binding [106]. Others have suggested [94] that there is a second (inhibitory) binding site in IDO and that this is the origin of the inhibition—this is also plausible and there is evidence for more than one binding site (or at least multiple binding conformations) [61, 109–112], including in a recent structure for human TDO where a second l-Trp binding site (referred to as an exo site) has been clearly observed at >40 Å from the active site [82].

## What goes around comes around: the lasting contribution of Osamu Hayaishi

Heme dioxygenases have floated into fashion, out of it, and back in again. The early contributions that Hayaishi made to the dioxygenase field are a lasting legacy that form a framework of reference to this day and will serve us all well as the field moves to the future.

## Footnotes

1 Fred Sanger was Neuberger’s first Ph.D. student.
